# Supplementary material for: Fitness cost associated with cell phenotypic switching drives population diversification dynamics and controllability
Source: Nat Commun. 2023 Oct 2;14:6128. doi: 10.1038/s41467-023-41917-z (PMC10545768; doi:10.1038/s41467-023-41917-z)
Supplement: Supplementary file 1 — Supplementary information [file 41467_2023_41917_MOESM1_ESM.pdf]

Fitness cost associated with cell phenotypic switching drives population diversification dynamics and controllability

Henrion *et al.*

Supplementary Note 1. Determination of the entropy (H) of the population from automated FC data and reproducibility of these data

Information theory has been used in this work to characterize the response of cell populations to environmental perturbations. This framework involves the computation of entropy H, which can be regarded as a measurement of uncertainty about the response of the cell population (output) in function of the environmental stimulation (input). This input-output relationship, which is the basis of information theory, will be detailed in Supplementary Note 3. In this note, we'll concentrate on the description of the entropy H as a measurement of the level of heterogeneity of the population. Entropy can be considered as a measure of uncertainty about the outcome of a draw from a probability distribution[1]. As an example, if we pick randomly a cell in our population, how much are we surprised to pick one cell with a given GFP level? In our case, we can measure the entropy based on fluorescence distribution acquired with automated FC based on the following equation:

$$H = -\sum_{i=1}^{m} p(x_i).\log_2 p(x_i) \qquad \textbf{(1)}$$

With m being the number of states observed (i.e., GFP classes in our cases) and p being the probability to observe this state. The probability to observe different classes of fluorescence can be easily determined based on automated FC. The computation of H has been exemplified based on fictive population of cells clustered in three fractions according to the level of GFP exhibited by cells (Supplementary Figure 1). As an example, the computation for the first population distribution (Supplementary Figure 1a) is performed as $H = -[(0.1.\log_2 0.1) + (0.6.\log_2 0.6) + (0.3.\log_2 0.3)] = 1.29$ bits.

Based on this first example, the entropy of the population can be either increased (Supplementary Figure 1b), the maximum entropy value being reached when cells are equally distributed into the 3 different clusters. On the opposite, H can be decreased and set to zero when all the cells exhibit the same fluorescence range (Supplementary Figure 1c). This approach has been applied to automated FC data (animated movies of the automated FC data for all the biological systems considered are available at GitLab [https://gitlab.uliege.be/mipi/published-software/mipi-model-and-simulation-database/] for computing the evolution of H(t) for different types of cell population (Supplementary Figure 2 for three out of six of the cell systems investigated). In this case, we applied 50 bins for the computation of H(t). Reproducibility of the corresponding experiments conducted in Segregostat is provided in Supplementary Figure 3.

Supplementary Note 2. Determination of the fitness cost associated with phenotypic switching

For the systems exhibiting high switching cost, the quantification of this parameter was a bit difficult because cells switched stochastically from the OFF state to the ON state. This was particularly true for the P_{glc3}:GFP system in yeast where only a small fraction of cells decided to switch under normal cultivation conditions. This effect can be observed based on MSCC experiments run at different glucose concentration (Supplementary Figure 4). However, the determination of the switching cost was easier in the case of the pET28 (T7 polymerase) system in *E. coli* BL21(DE3), this system being inducible upon addition of lactose. Accordingly, we cultivated systems exhibiting low (P_{araB}:GFP and P_{lacZ}:GFP in *E. coli* W3110) and high (pET28 system in *E. coli* BL21(DE3)) in a multiplate cultivation device (Biolector) (Supplementary Figure 5). Based on these data, it can be observed that the effect of gene circuit activation itself and the change of carbon source are coupled most of the time. However, we can also see that, in most of the cases, the activation of the gene circuit itself drives the switching cost. It can be seen that the *E. coli* W3110 strain exhibit slightly reduced growth when cultivated on lactose and arabinose, independently of the presence of a fluorescent reporter. On the opposite, we can see that the *E. coli* BL21(DE3) strains exhibit a huge reduction in growth upon cultivation on lactose, mostly due here to the activation of the chromosomal insert of the T7 RNA polymerase.

Supplementary Note 3. Experimental determination of the response function for the P_{araB}::GFP system in *E. coli* and the P_{glc3}::GFP system in *S. cerevisiae* and computation of the mutual information (MI)

Information theory relies on the characterization of the input-output relationship for various systems, and has been applied recently to the analysis of signal propagation in biological systems[23]. Basically, MI allows to quantify how much we can know about an input (e.g., change in environmental condition) from the output (i.e., in our case, the fluorescence distribution of the population). The first step for the computation of MI is to calculate the entropy of the population exposed to defined environmental conditions. These conditional distributions represent the response function of our cellular systems, and more precisely the P_{araB}:GFP system in *E. coli* (Supplementary Figure 6) and P_{glc3}:GFP system in *S. cerevisiae* (Supplementary Figure 7).

Characterization of the response function for the *S. cerevisiae* P_{glc3}:GFP system

The response function of the P_{glc3}:GFP in *S. cerevisiae* was determined by growing culture at different dilution rates in chemostat (Supplementary Figure 7). For this purpose, the dilution rate of a chemostat was progressively increased to release the stress response of the population. This procedure is known as accelerostat (A-stat). In our case, the pump flow rate was modified with a step change every 2 hours, resulting in a progressive increase of the dilution rate of 0.002 h^{-1} per hour. This incremental range was chosen in order to ensure pseudo steady-state for each increment. The entire process was followed by automated FC for mapping the GFP distribution of the cell population (Supplementary Figure 7).

Computation of mutual information (MI) based on the response function of a cell population

Knowing the response function, and the corresponding conditional GFP distribution, it is possible to compute the MI of a specific cellular system. This computation will be exemplified for the P_{glc3}::GFP reporter in *S. cerevisiae*. The environmental input for this system is the glucose uptake rate determined based on the value of the dilution rate, as well as based on glucose and biomass measurement. The conditional fluorescence distributions were then acquired for different substrate uptake rates and H was computed accordingly (Supplementary Figure 8a). If all the fluorescence distributions are summed up, the corresponding entropy value is the total entropy of the system. MI is then computed according to (Supplementary Figure 8b):

$$\mathrm{MI}(y,x) = \mathrm{H}(x) - \mathrm{H}(x,y) \qquad (2)$$

With H(x) being the total entropy for output x and H(x,y) being the conditional entropy computed from the conditional distribution of the output x (x, being GFP distribution and y being the sugar uptake rate).

When doing so, it is important to adjust the number of bins used for computing the entropy. In our case, this number was set to 50 bins and leads to a precise computation of MI without increasing the computing power (Supplementary Figure 9).

In order to explain the differences in controllability between the different systems investigated, we computed the mutual information (MI) between the environmental conditions and the activation of the target gene circuit for the P_{araB}::GFP and P_{glc3}::GFP systems. MI is a proxy derived from information theory[26,27] and involves the computation of the entropy of the cell

population, as defined in the previous section. In short, MI tells us how much we can learn about the input (i.e., in our case the environmental stimulus used for entraining the cell population) from the output (i.e., in our case the distribution of GFP in cell population, the dispersion being quantified based on the entropy). Thus, in our case, MI is a proxy for information transfer efficiency between the inducer concentration and the cell population induction. The total entropy for each system was evaluated by summing up all the conditional probabilities obtained by exposing cell populations to different cultivation conditions (Supplementary Figure 10). MI was obtained by subtracting the time-dependent entropies to the total entropies recorded for each system. For the P_{araB}:GFP system, MI is already relatively high in the chemostat, leaving little room for improvement in the Segregostat (Supplementary Figure 10). It means that a reduction in entropy between the two cultivation modes has to be expected when the amount of information conveyed in chemostat condition is low (e.g., when cells cultivated in chemostat do not sense the inducer and, accordingly, do not activate the corresponding gene circuit). This is exactly what happened during the chemostat culture of the P_{glc3}::GFP system (Supplementary Figure 10d). MI analysis had pointed out that there was still room for additional reduction in entropy (Supplementary Figure 10), and this was observed in Segregostat where glucose pulses reduced the average entropy (Supplementary Figure 10).

Supplementary Note 4. Flow cytometry Stochastic Kinetic Simulator (FlowStocKS)

FlowStocKS is a computational tool designed to simulate the phenotype distributions of a microbial population under different environmental regimes (Chemostat and Segregostat). Following the model framework classification proposed by Hartmann *et al.*[5], FlowStocKS can be considered as: i) biologically segmented as it considers single cells; ii) abiotically unsegmented as it assumes a homogeneous environment; iii) an unstructured cell model as it does not take intracellular kinetics or metabolic fluxes into consideration. FlowStocKS comprises two modules: the growth module and the switch module. The system is resolved using a Markov chain with discrete time, where the growth module is described by a set of ordinary differential equations (ODE). These ODEs detail how single cells grow (Equation **3** and **4**) and consume (Equation **5**) their substrate (S), in accordance with the Monod-type equations that include a non-competitive growth inhibition term.

$$\mu = \mu_{\max}.\frac{[S]}{[S]+Ks}\frac{KI}{[I]+KI} \quad \textbf{(3)}$$

$$\frac{dX}{dt} = \mu.X - D.X \quad \textbf{(4)}$$

$$\frac{dS}{dt} = D.[S_{Feed}] - Y.\mu.X - D.[S] \quad \textbf{(5)}$$

Where:

μ_{max} = Maximal growth rate

μ = Growth rate

X = Cell biomass

KI = Growth inhibition constant

Ks = Affinity for the substrate

Y = Substrate to biomass yield

D = Dilution rate

$[S_{Feed}]$ = Substrate concentration in the feed

$[I]$ = Inhibitor concentration

$[S]$ = Substrate concentration

t = Time

In the growth module, single cells are simulated to grow until they double in size, at which point they divide into two daughter cells. Additionally, to simulate continuous cultivations, cells are randomly flushed out of the system based on a probability (P_{out}) set by the dilution rate and the time step (T_{step}) used in the simulation (Equation **6**).

$$P_{out} = D \,.\, T_{step} \quad \textbf{(6)}$$

The growth parameters (μ_{max}, KI, Y, Ks) are given by the switch module and define the phenotype of each cell. To initiate the switching process, a cell must first cross a time threshold by accumulating T_{step}, and this commitment process is governed by a switching probability (P). This probability is determined by a response function, taking the inducer concentration (i) as input. The P function is a classical sigmoid function characterized by a steepness (n) and a 50% switching probability at concentration (K).

$$P(i) = \frac{[i]^n}{[i]^n + K} \quad (7)$$

The accumulation of T_{step} is analogous to the build-up of enzymes that are necessary for the expression of a different phenotype, commonly known in many processes e.g., substrate consumption switching and the diauxic shift time[678]. Once the time threshold (τ) is reached, the cell switches phenotype. In our experimental set-up, we use GFP-based reporters to track the phenotype switch. Thus, once the time threshold is reached a GFP is produced as a burst following a production rate set by a gamma distribution ($\Gamma_{(q_{gfp},2)}$)[9]. Similarly to Taniguchi *et al.*[9], we assumed that active degradation of GFP or the inhibitor is neglectable and accordingly dilution is determined by cell division.

With FlowStocKS, we conducted simulations using a constant time step (T_{step}) of 1 minute, which is two orders of magnitude faster than the fastest process considered (T_{step}<<< μ_{max}). In these simulations, we modeled a population of 10,000 cells, each with an initial mass equal to 1/1000th of the initial biomass. Our simulations were based on the growth characteristics of *Saccharomyces cerevisiae* (Supplementary Table 3). The switch to the stressed phenotype is triggered by glucose limitation resulting in growth inhibition; the latter is the fitness cost. In the simulations, the fitness cost is the reduction in growth rate of the cell that switches and produces GFP compared to a cell that does not switch. To simplify our analysis, we assumed that the resulting GFP production is linearly correlated with the inhibitor concentration, and thus its concentration was used as the inhibitor.

Therefore, by varying inhibition value KI (lower value means higher growth inhibition), we were able to compute the phenotype distribution both in Chemostat and Segregostat for different fitness cost associated to switching. All FlowStocKS codes are available at GitLab [https://gitlab.uliege.be/mipi/published-software/2023-henrion].

Supplementary Table 1. Sequences and primers used in this work.

**Primer name**	**Sequence (5'-3')**
Fw_sgRNA_20N_Ara	agctagctcagtcctaggtataatactagtCGCCCGCAGGATATTCGTCGgttttagagctagaaatagcaagttaaaa
Fw_fragment1	gttaaacgagtatcccggcagca
Rv_fragment1	cgtttcactccatccaaaaaaacgg
Fw_fragment2	tggagtgaaacgtgactgtataaaaccacagccaa
Rv_fragment2	catcggcctcgtagacggtaac

Supplementary Table 2. Operating conditions used for conducting the chemostat and Segregostat experiments.

Strain	Process conditions	Control conditions
E. coli W3110 $P_{araB:}$:*GFPmut2*	D = 0.45 h^{-1} pH = 7 T = 37 °c<br>Aeration = 1 L/min<br>Agitation = 1000 min^{-1}<br>[Glucose feed] = 5 g/L<br>$[\text{Arabinose feed}]_{chemostat}$ = 1.5 g/L<br>$[\text{Arabinose feed}]_{Segregostat}$= 0	Chemostat: none<br>Segregostat:<br>Regulation threshold = 50 % cells bellow 1000 F.U.<br>Regulation = Pulse 0.15 g arabinose
E. coli W3110 $P_{lacZ:}$:*GFPmut2*	D = 0.45 h^{-1} pH = 7 T = 37 °c<br>Aeration = 1 L/min<br>Agitation = 1000 min^{-1}<br>[Glucose feed] = 5 g/L<br>$[\text{Lactose feed}]_{chemostat}$ = 1.5 g/L<br>$[\text{Lactose feed}]_{Segregostat}$= 0	Chemostat: none<br>Segregostat:<br>Regulation threshold = 50 % cells bellow 1000 F.U.<br>Regulation = Pulse 0.15 g lactose
E. coli BL21 pET28:GFP	D = 0.45 h^{-1} pH = 7 T = 37 °c<br>Aeration = 1 L/min<br>Agitation = 1000 min^{-1}<br>[Glucose feed] = 5 g/L<br>$[\text{Lactose feed}]_{chemostat}$ = 1 g/L<br>$[\text{Lactose feed}]_{Segregostat}$= 0	Chemostat: none<br>Segregostat:<br>Regulation threshold = 50 % cells bellow 1000 F.U.<br>Regulation = Pulse 0.5 g lactose
E. coli W3110 P_{bolA}:*GFPmut2*	D = 0.45 h^{-1} pH = 7 T = 37 °c<br>Aeration = 1 L/min<br>Agitation = 1000 min^{-1}<br>[Glucose feed] = 5 g/L	Chemostat: none<br>Segregostat:<br>Regulation threshold = 50 % cells above 2000 F.U.<br>Regulation = Pulse 0.2 g glucose
B. subtilis 168 P_{spoIIE}:GFPmut2	D = 0.1 h^{-1} pH = 7 T = 37 °c<br>Aeration = 1 L/min<br>Agitation = 1000 min^{-1}<br>[Glucose feed] = 5 g/L	Chemostat: none<br>Segregostat:<br>Regulation threshold = 20% cells above 1000 F.U.<br>Regulation = Pulse 0.2 g glucose
S. cerevisiae CEN.PK 113-7D P_{glc3}:eGFP	D = 0.1 h^{-1} pH = 5 T = 30 °c<br>Aeration = 1 L/min<br>Agitation = 1000 min^{-1}<br>[Glucose feed] = 5 g/L	Chemostat: none<br>Segregostat:<br>Regulation threshold = 50% cells above 5000 F.U.<br>Regulation = Pulse 0.2 g glucose

Supplementary Table 3. Parameters used for running FlowStocKS simulations.

Parameter	Description	Value	Unit	Source
µ_max_glucose	Maximal growth rate on glucose	0.54	h^{-1}	[10]
Ks_glucose	Affinity constant for glucose	0.034	g/L	[10]
Y_glucose	Substrate to biomass yield for glucose yeast	0.5	g/g	[11]
n	Hill coefficient	-2		This study[a]
k	50 % probability switching concentration	0.05	g/L	This study[a]
Gfp_prod	Mean value of GFP production burst given a gamma distribution of scale 2 and growth inhibition	1e5	fu	This study[b] by $\frac{[I]}{[I]+KI}$
KI	Growth inhibition	From 0 to 1e99	fu	
delay (τ)	Time delay between pulse and GFP production	0.4	h	This study[c]

[a]Approximated from the proportion of induced cells for different glucose concentrations observed in microfluidic cultivation.
[b]Set to evaluate the impact of inhibition strength, Gfp_prod and KI individual values are arbitrary, only the ratio matters as the inhibition strength is set.
[c]A delay of 0.4 hours (24 minutes) between sensing and switching was selected based on Segregostat observations, where two consecutive automated FC measurements (24 minutes) separate a pulse and the first increase in fluorescence.

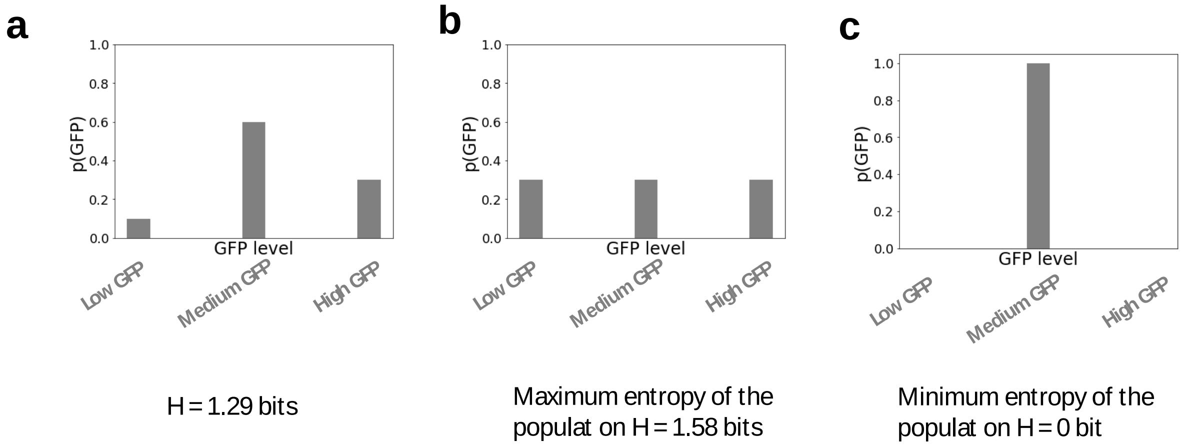

Supplementary Figure 1. Examples of computation of the entropy H for a cell population clustered in three bins (computation according to Equation **1**). Each bin corresponds to a subpopulation of cells with a given fluorescence range i.e., low, medium or high, leading to different level of entropy H with **a** medium level of H, **b** maximum level of H for a three states system and **c** minimum level of entropy H.

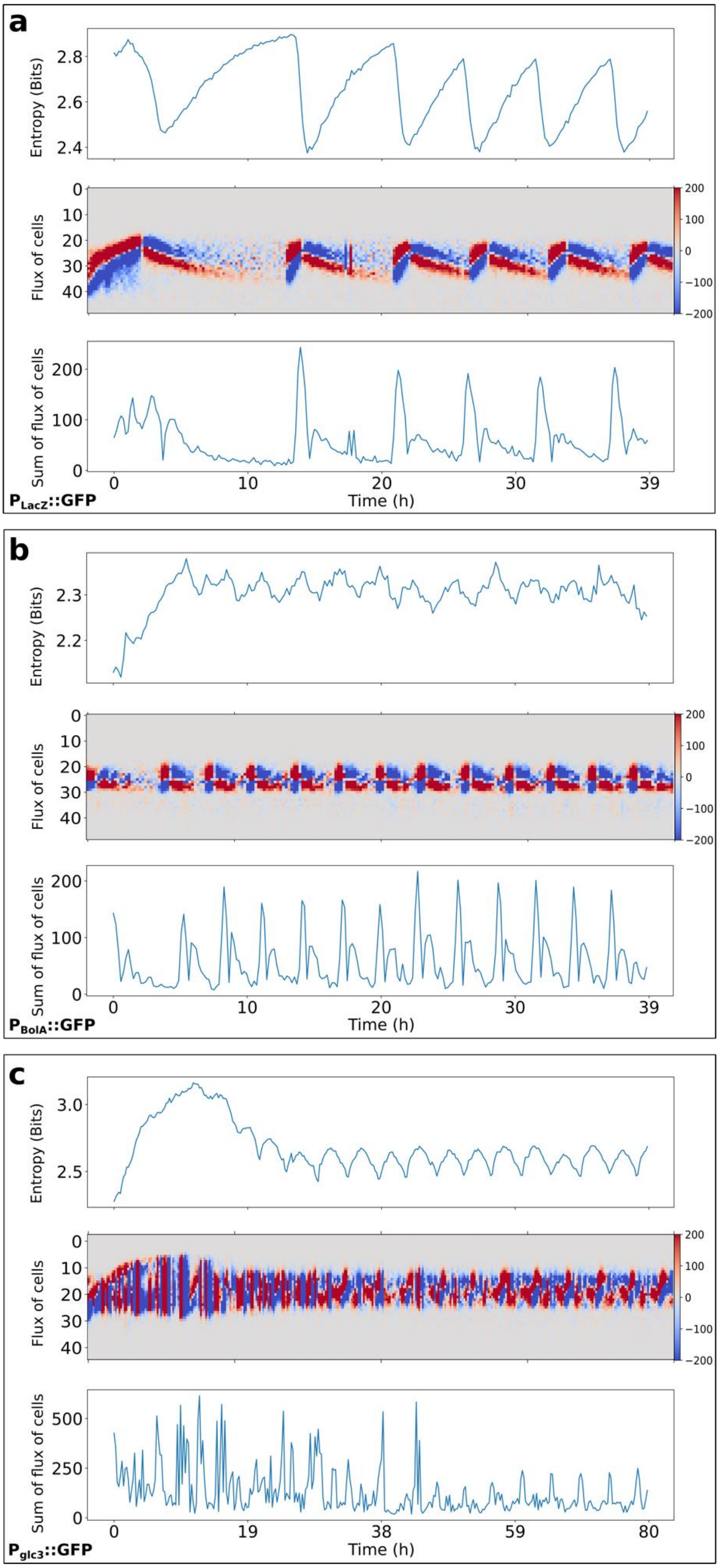

Supplementary Figure 2. Computation of the entropy and flux of cells for different cellular systems. **a** P_{lacZ}::GFP (*E. coli*), **b** P_{bolA}::GFP (*E. coli*) and **c** P_{glc3}::GFP (*S. cerevisiae*).

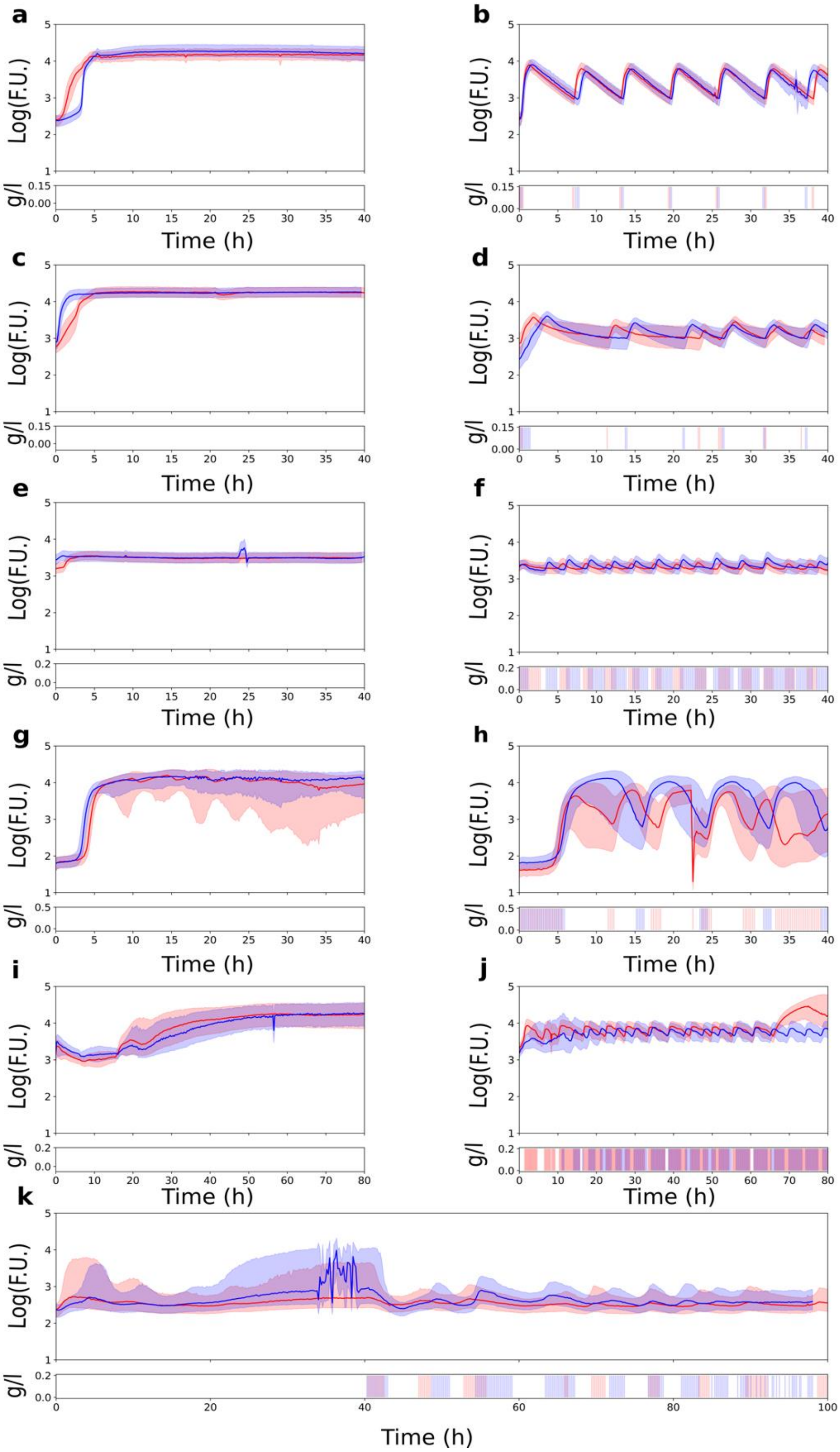

Supplementary Figure 3. Assessment of the reproducibility of the Segregostat experiments. Evolution of the median fluorescence (plain line) and interquartile range (shadowed area around the plain line) between two biological replicates for **a** *E. coli* P_{araB}::GFP in chemostat and **b** in Segregostat. **c** *E. coli* P_{lacZ}::GFP in chemostat and **d** in Segregostat. **e** *E. coli* P_{bolA}::GFP in chemostat and **f** in Segregostat. **g** *E. coli* P_{lacZ}::GFP in chemostat and **h** in Segregostat. **i** *S. cerevisiae* P_{glc3}::GFP in chemostat and **j** in Segregostat. **k** *B. subtilis* P_{spoIIE}::GFP in chemostat for 40 h followed by Segregostat.

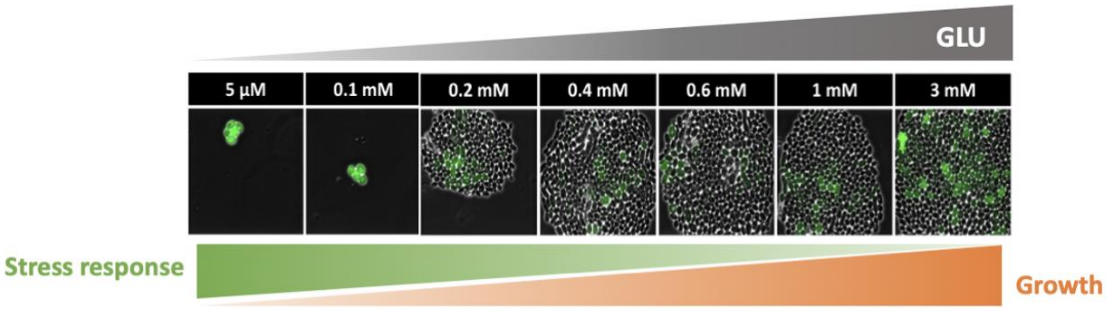

Supplementary Figure 4. Pictures of yeast P$_{glc3}$::GFP microcolonies cultivated in a MSCC device at different glucose concentrations. Movies of a microcolony growing at a concentration of 0.1 mM and 1mM can be accessed in Supplementary Movie 1 and 2, respectively).

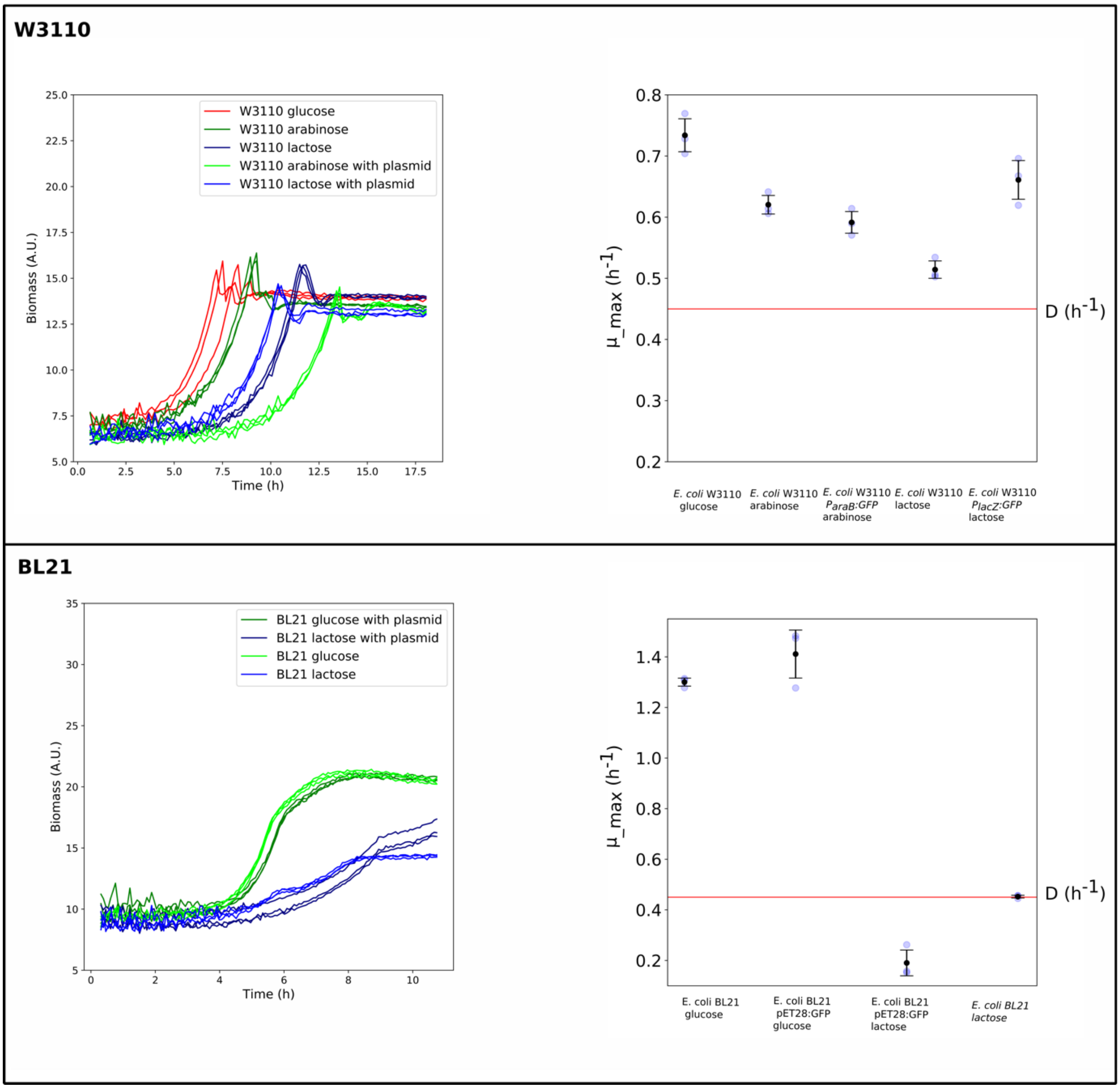

Supplementary Figure 5. Experimental evaluation of the switching cost. Growth of different strains, the maximal growth rate of each triplicate is presented as a blue dot (plots on the right) and the means and standard deviations as a black dot with error bar. (Top) Determination of the maximal growth rate from Biolector cultivation with automated biomass measurement (n=3) for *E. coli* W3110, *E. coli* W3110 P_{araB}::GFP and *E. coli* W3110 P_{lacZ}::GFP on glucose, arabinose and lactose as carbon sources. (Bottom) Determination of the maximal growth rate from Biolector cultivation with automated biomass measurement (n=3) for *E. coli* BL21 (DE3) pET28::GFP on glucose and lactose and *E. coli* BL21 (DE3) on glucose and lactose. The red horizontal line is the dilution rate (0.45 h^{-1}) used for the continuous cultivations.

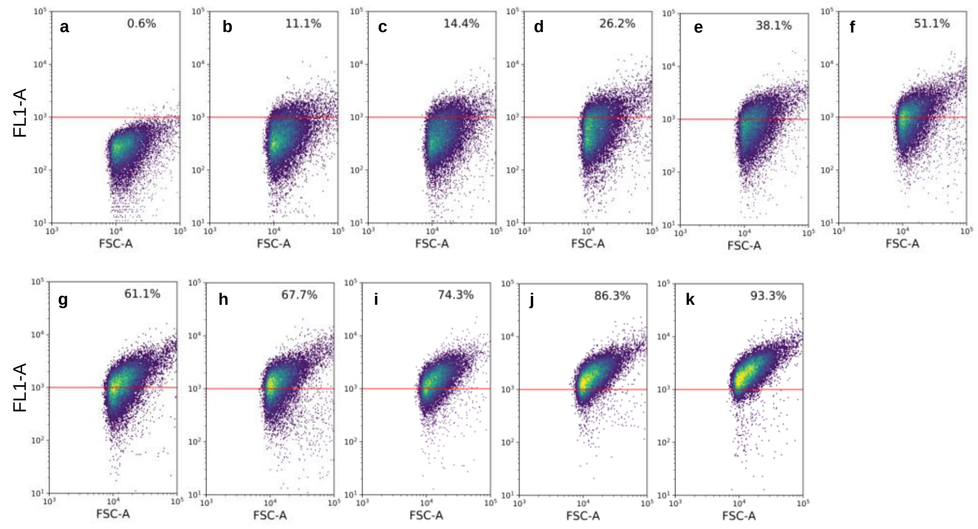

Supplementary Figure 6. Analysis of the input-output relationship for *E. coli* P_{araB}:GFP. Scatter plots of cell size (FSC-A) versus GFP fluorescence (FL1-A) for *E. coli* W3110 *ΔaraBAD* P_{araB}::GFPmut2 exposed to arabinose concentrations of **a** 0, **b** 0.025, **c** 0.05, **d** 0.1, **e** 0.15, **f** 0.20, **g** 0.25, **h** 0.30, **i** 0.50, **j** 1.00 and **k** 2.00 g/L. The red line stands for the fluorescence threshold (i.e., 1000 F.U.) used for computing the GFP positive fraction of cells. Each FC analysis comprises 20,000 analyzed cells. The experiment was repeated twice with the similar results.

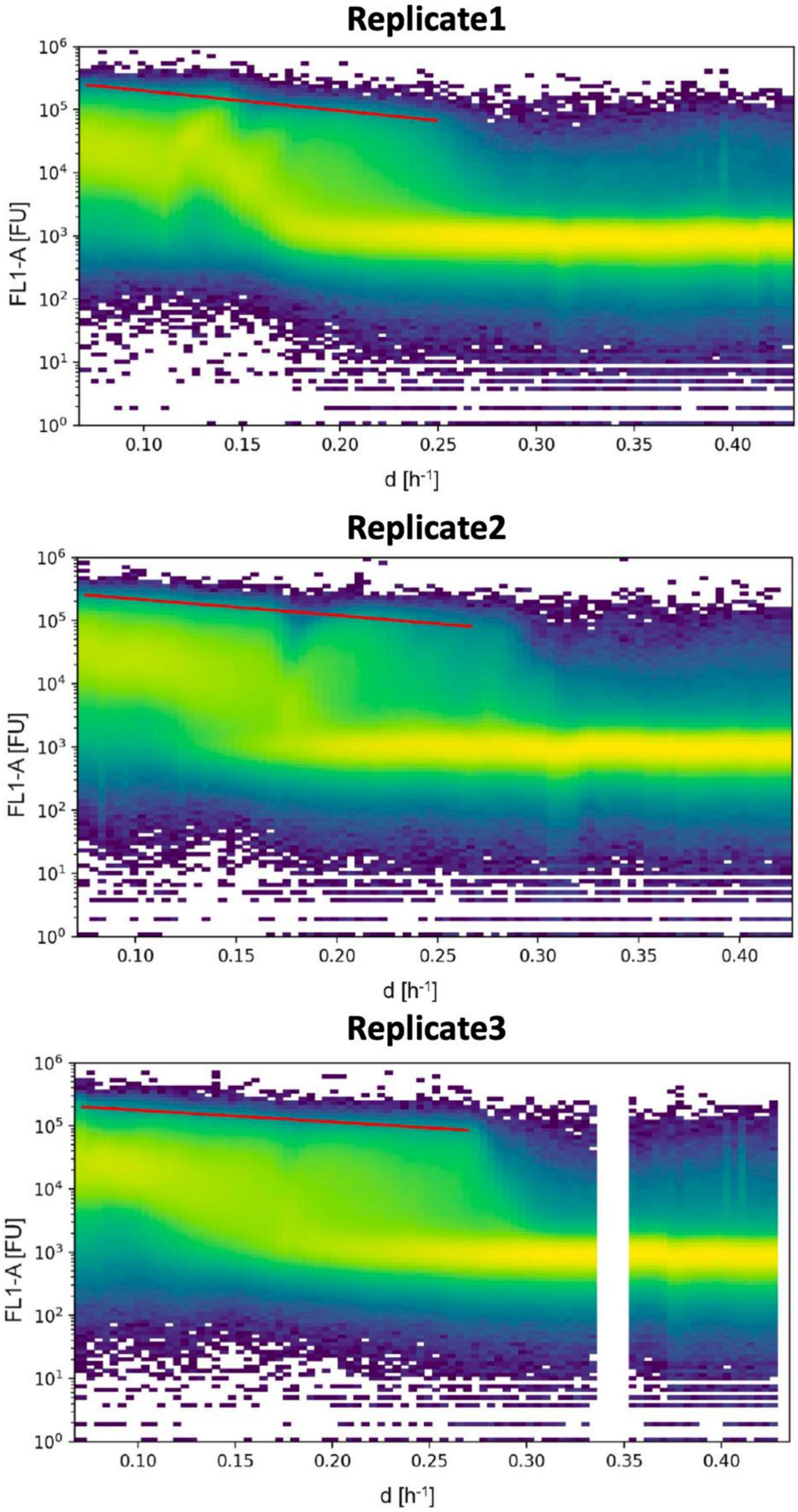

Supplementary Figure 7. Analysis of the input-output relationship for *S. cerevisiae* P_{glc3}:GFP. A-stat experiment monitored based on automated FC. The GFP level distribution (FL1-A channel) was determined for each dilution rate (D). The red line highlights the progressive release of the stress response at the population level based on the deactivation of the P_{glc3}::GFP reporter. Each FC analysis comprises 20,000 analyzed cells, the cultivation was performed in triplicate with similar outcomes (the three biological replicates are displayed).

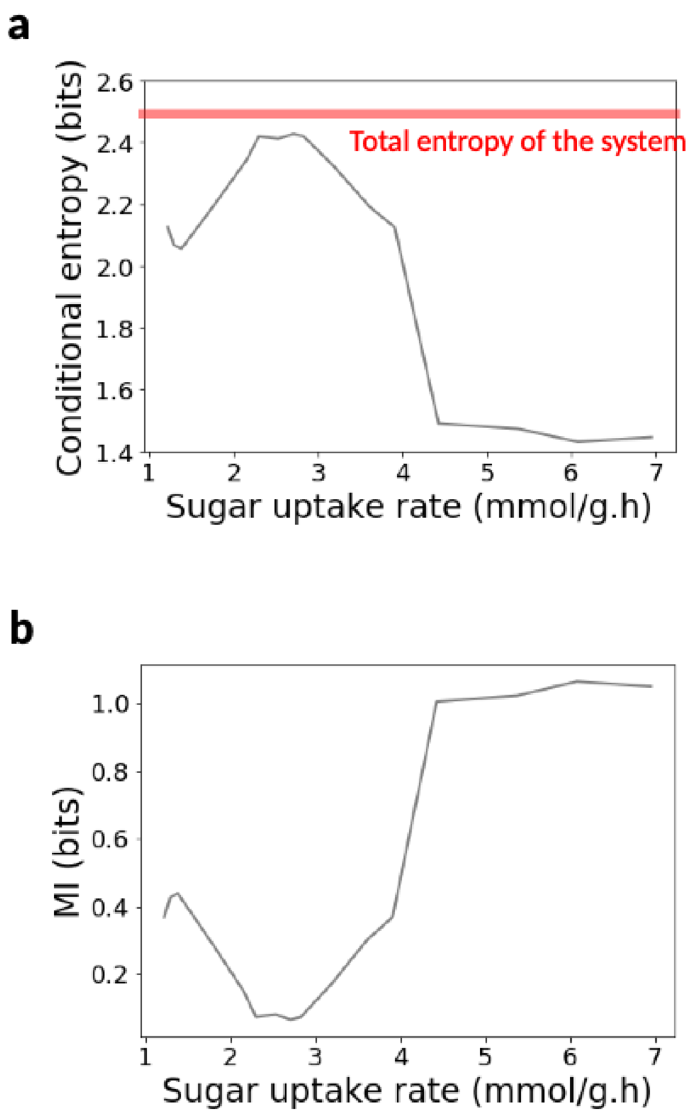

Supplementary Figure 8. Evaluation of the mutual information (MI). **a** Evolution of the conditional entropy for the P_{glc3}:GFP in *S. cerevisiae* exposed to different uptake rates. **b** MI can be deduced by subtracting the value of the total entropy of the system (2.49 bits in this case) by the corresponding conditional entropy.

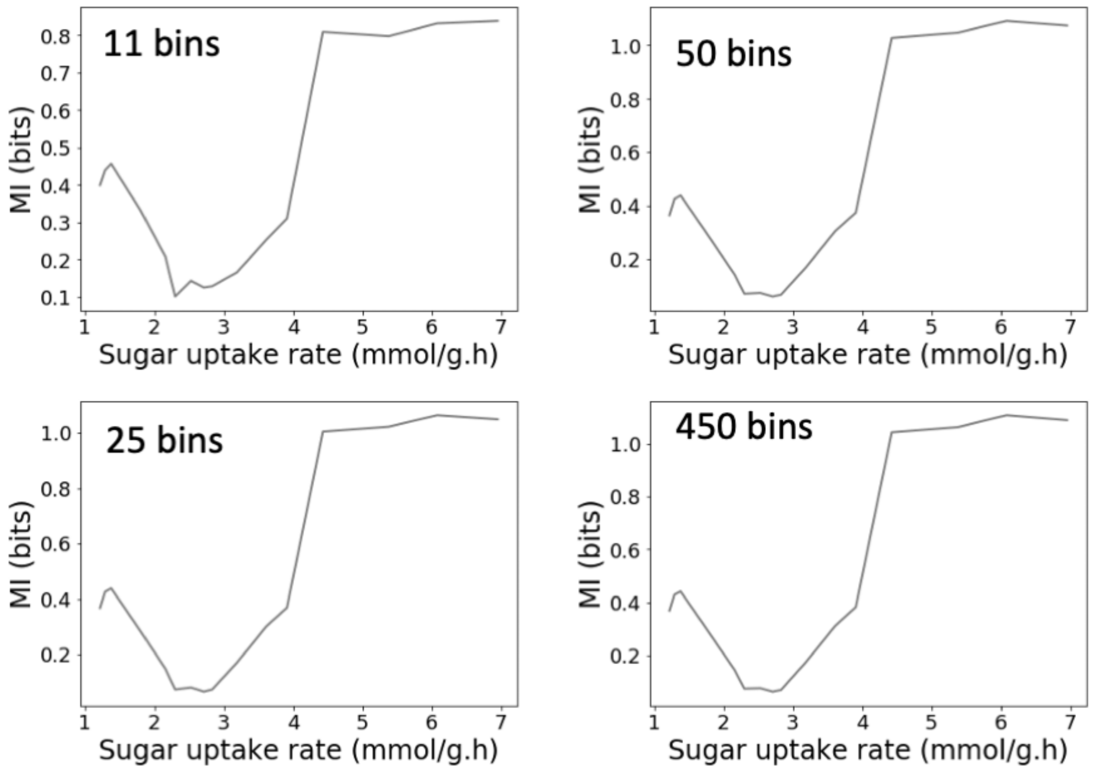

Supplementary Figure 9. Evaluation of the mutual information (MI). Impact of the binning procedure (number of bins considered) on the estimation of MI for the P_{glc3}::GFP in *S. cerevisiae* (data extracted from A-stat experiments).

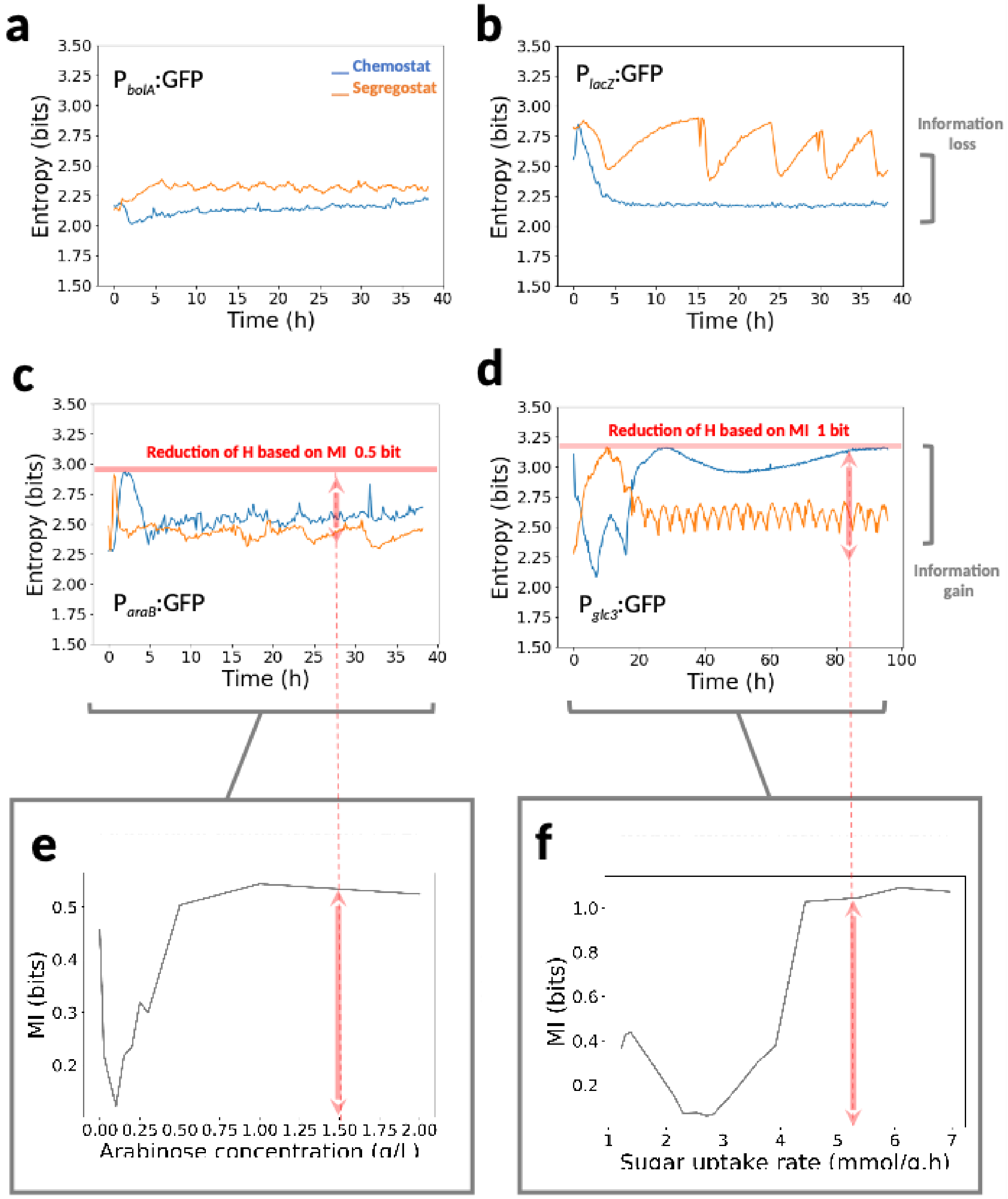

Supplementary Figure 10. Comparative analysis of the entropy computed from the binned population data for four different cell systems. Comparative analysis of the entropy profile for the **a** P_{bolA}:GFP system in *E. coli*. **b** P_{lacZ}:GFP system in *E. coli*. **c** P_{araB}::GFP system in *E. coli*. **d** P_{glc3}::GFP system in *S. cerevisiae*. For the P_{araB}:GFP and P_{glc3}:GFP systems, the conditional probabilities, i.e., the GFP distribution of the population exposed at different environmental conditions, have been experimentally determined (Supplementary Note 2), allowing the computation of the mutual information (MI). **e** MI for the P_{araB}:GFP system exposed to different arabinose concentrations. The MI distribution for the P_{araB}:GFP system suggests that, at high arabinose concentration, a gain of information of approximately 0.5 bit has to be expected (the value has been reported by a red line on Figure 3C). **f** MI for the P_{glc3}:GFP system exposed to different sugar uptake rates in a accelerostat cultivation device (Supplementary Figure 4). The MI distribution for the P_{glc3}:GFP system suggests that, at high glucose concentration a gain of information of approximately 1 bit has to be expected (the value has been reported by a red line on Figure 3d).

Supplementary references

1. Harte, J. & Newman, E. A. Maximum information entropy: a foundation for ecological theory. *Trends Ecol Evol* **29**, 384–389 (2014).
2. Cheong, R., Rhee, A., Wang, C. J., Nemenman, I. & Levchenko, A. Information transduction capacity of noisy biochemical signaling networks. *Science* **334**, 354–358 (2011).
3. Hansen, A. S. & O'Shea, E. K. Limits on information transduction through amplitude and frequency regulation of transcription factor activity. *eLife* **4**, (2015).
4. Jiang, Y. *et al.* Multigene editing in the *Escherichia coli* genome via the CRISPR-Cas9 system. *Appl Environ Microbiol* **81**, 2506–2514 (2015).
5. Hartmann, F. S. F., Udugama, I. A., Seibold, G. M., Sugiyama, H. & Gernaey, K. V. Digital models in biotechnology: Towards multi-scale integration and implementation. *Biotechnol Adv* **60**, 108015 (2022).
6. Chu, D. & Barnes, D. J. The lag-phase during diauxic growth is a trade-off between fast adaptation and high growth rate. *Sci Rep* **6**, 25191 (2016).
7. Martinez, J. A. *et al.* Controlling microbial co-culture based on substrate pulsing can lead to stability through differential fitness advantages. *PLoS Comput Biol* **18**, e1010674 (2022).
8. Jones K. D., K. D. S. Cybernetic model of the growth dynamics of *Saccharomyces cerevisiae* in batch and continuous cultures. *Journal of Biotechnology* **71**, 105–131 (1999).
9. Taniguchi, Y. *et al.* Quantifying *E. coli* proteome and transcriptome with single-molecule sensitivity in single cells. *Science* **329**, 533–538 (2010).
10. Jain, V. K. Relationship between energy metabolism and growth. I. Glucose dependence of the exponential growth rate of *Saccharomyces cerevisiae*. *Arch Mikrobiol* **72**, 252–259 (1970).
11. Milo, R. & Phillips, R. *Cell Biology by the Numbers*. (Garland Science, 2016).
