# Supplementary material for: Fitness cost associated with cell phenotypic switching drives population diversification dynamics and controllability
Source: Nat Commun. 2023 Oct 2;14:6128. doi: 10.1038/s41467-023-41917-z (PMC10545768; doi:10.1038/s41467-023-41917-z)
Supplement: Supplementary file 3 — Description of Additional Supplementary Files [file 41467_2023_41917_MOESM3_ESM.pdf]

Description of Additional Supplementary Files

File Name: Supplementary Movie 1
Description: Cultivation of *S. cerevisiae* carrying a P_{glc3}::GFP reporter in a MSCC device. Chemically defined medium is constantly perfused into the chamber with a glucose concentration of 0.1mM, leading to a drastic reduction in growth of the colony and full activation of the P_{glc3}::GFP reporter.

File Name: Supplementary Movie 2
Description: Cultivation of *S. cerevisiae* carrying a P_{glc3}::GFP reporter in a MSCC device. Chemically defined medium is constantly perfused into the chamber with a glucose concentration of 1mM.

File Name: Supplementary Movie 3
Description: Cultivation of *S. cerevisiae* carrying a P_{glc3}::GFP reporter in a dMSCC device. Cultivation conditions are periodically switched between chemically defined media containing glucose at a concentration of 0.1 and 1 mM respectively. The feast-to-famine transitions have been applied with predefined durations i.e., $T_{0.1mM}$ = 0.8 h and T_{1mM} = 3h, for mimicking the Segregostat conditions.

File Name: Supplementary Movie 4
Description: FlowStocKS simulations of chemostat cultivations under variable fitness cost.
